# (*E*)-1-{4-[Bis(4-fluoro­phen­yl)meth­yl]piperazin-1-yl}-3-(4-eth­oxy­phen­yl)prop-2-en-1-one

**DOI:** 10.1107/S160053681104801X

**Published:** 2011-11-16

**Authors:** Yan Zhong, XiaoPing Zhang, Bin Wu

**Affiliations:** a School of Chemistry and Chemical Engineering, Southeast University, Sipailou No. 2 Nanjing, Nanjing 210096, People’s Republic of China; b Centre of Laboratory Animals, Nanjing Medical University, Hanzhong Road No. 140 Nanjing, Nanjing 210029, People’s Republic of China; c School of Pharmacy, Nanjing Medical University, Hanzhong Road No. 140 Nanjing, Nanjing 210029, People’s Republic of China

## Abstract

In the title mol­ecule, C_28_H_28_F_2_N_2_O_2_, the ethene bond exhibits an *E* conformation and the piperazine ring adopts a chair conformation. The amide-N atom of the piperazine ring is almost planar (bond-angle sum = 358.8°) whereas the other N atom is clearly pyramidal (bond-angle sum = 330.5°). The dihedral angle between the fluoro­benzene rings is 76.36 (17)°. In the crystal, inversion dimers linked by pairs of C—H⋯O hydrogen bonds generate *R* ^2^_2_(22) loops.

## Related literature

For a related structure and background to cinnamic acid derivatives, see: Zhong & Wu (2011 ▶). For further synthetic details, see: Wu *et al.* (2008 ▶).
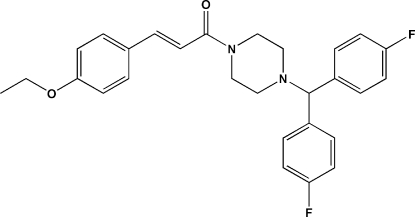


## Experimental

### 

#### Crystal data

C_28_H_28_F_2_N_2_O_2_
*M* *_r_* = 462.52
Monoclinic, 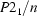

*a* = 10.227 (2) Å
*b* = 11.897 (2) Å
*c* = 20.149 (4) Å
β = 100.04 (3)°
*V* = 2414.0 (8) Å^3^
*Z* = 4
Mo *K*α radiation
μ = 0.09 mm^−1^
*T* = 293 K
0.30 × 0.20 × 0.20 mm

#### Data collection

Enraf–Nonius CAD-4 diffractometer
4698 measured reflections
4436 independent reflections
2454 reflections with *I* > 2σ(*I*)
*R*_int_ = 0.037
3 standard reflections every 200 reflections  intensity decay: 1%

#### Refinement

*R*[*F* ^2^ > 2σ(*F* ^2^)] = 0.060
*wR*(*F* ^2^) = 0.187
*S* = 1.00
4436 reflections
307 parameters
8 restraints
H-atom parameters constrained
Δρ_max_ = 0.58 e Å^−3^
Δρ_min_ = −0.24 e Å^−3^

### 

Data collection: *CAD-4 EXPRESS* (Enraf–Nonius, 1994 ▶); cell refinement: *CAD-4 EXPRESS*; data reduction: *XCAD4* (Harms & Wocadlo, 1995 ▶); program(s) used to solve structure: *SHELXS97* (Sheldrick, 2008 ▶); program(s) used to refine structure: *SHELXL97* (Sheldrick, 2008 ▶); molecular graphics: *SHELXTL* (Sheldrick, 2008 ▶); software used to prepare material for publication: *SHELXL97* (Sheldrick, 2008 ▶).

## Supplementary Material

Crystal structure: contains datablock(s) I, New_Global_Publ_Block. DOI: 10.1107/S160053681104801X/hb6495sup1.cif

Structure factors: contains datablock(s) I. DOI: 10.1107/S160053681104801X/hb6495Isup2.hkl

Additional supplementary materials:  crystallographic information; 3D view; checkCIF report

## Figures and Tables

**Table 1:** Hydrogen-bond geometry (Å, °)

*D*—H⋯*A*	*D*—H	H⋯*A*	*D*⋯*A*	*D*—H⋯*A*
C9—H9*A*⋯O1^i^	0.93	2.60	3.422 (4)	148

Symmetry code: (i) 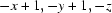.

**Fig. 1.**
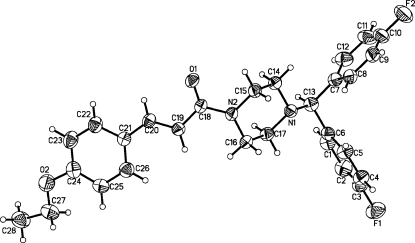
The molecular structure of (I) with displacement ellipsoids for non-H atoms drawn at the 70% probability level.

**Fig. 2.**
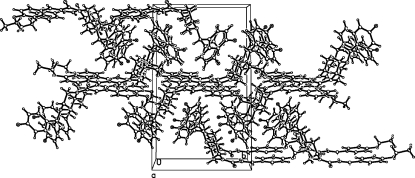
Packing diagram of the title compound.

## supplementary crystallographic information

### Comment

As part of our onging studies of compounds containing a cinnamoyl moiety (Zhong *et al.*, 2011), we now report the crystal structure of the title compound, (I). The molecule of the title compound exists an E conformation with respect to the C19=C20 ethene bond [1.332 (4)] and the torsion angle C18—C19—C20—C21 = 177.2 (3). The piperazine ring adopts a chair conformation with puchering parameters Q = 0.575 (3), Theta = 176.6 (3), Phi = 211 (6). The dihedral angle between the fluorobenzene rings is 76.36 (17). In the crystal, molecules are linked by intermolecular C—H···O interactions.

### Experimental

The synthesis follows the method of Wu *et al.* (2008). The title compound was prepared by stirring a mixture of (*E*)-3-(4-ethoxyphenyl)acrylic acid (0.769 g; 4 mmol), dimethyl sulfoxide (2 ml) and dichloromethane (30 ml) for 6 h at room temperature. The solvent was removed under reduced pressure. The residue was dissolved in acetone (15 ml) and reacted with 1-(bis(4-fluorophenyl)methyl)piperazine (1.730 g; 6 mmol) in the presence of triethylamine (5 ml) for 12 h at room temperature. The resultant mixture was cooled. The solid, (*E*)-1-(4-(bis(4-fluorophenyl)methyl)piperazin-1-yl)-3-(4- ethoxyphenyl)prop-2-en-1-one obtained was filtered and was recrystallized from ethanol.  Colourless blocks of (I) were grown in ethyl acetate by a slow evaporation at room temperature.

### Refinement

All non-hydrogen atoms were refined anisotropically. All hydrogen atoms were positioned geometrically with C—H distances ranging from 0.93 Å to 0.98 Å and refined as riding on their parent atoms with *U*ĩso~(H) = 1.2 or 1.5*U*~eq~ of the carrier atom.

### Crystal data

**Table:**

C_28_H_28_F_2_N_2_O_2_	*F*(000) = 976
*M**_r_* = 462.52	*D*_x_ = 1.273 Mg m^−^^3^
Monoclinic, *P*2_1_/*n*	Mo *K*α radiation, λ = 0.71073 Å
*a* = 10.227 (2) Å	Cell parameters from 25 reflections
*b* = 11.897 (2) Å	θ = 9–13°
*c* = 20.149 (4) Å	µ = 0.09 mm^−^^1^
β = 100.04 (3)°	*T* = 293 K
*V* = 2414.0 (8) Å^3^	Block, colorless
*Z* = 4	0.30 × 0.20 × 0.20 mm

### Data collection

**Table:**

Enraf–Nonius CAD-4 diffractometer	*R*_int_ = 0.037
Radiation source: fine-focus sealed tube	θ_max_ = 25.4°, θ_min_ = 2.0°
graphite	*h* = 0→12
ω/2θ scans	*k* = 0→14
4698 measured reflections	*l* = −24→23
4436 independent reflections	3 standard reflections every 200 reflections
2454 reflections with *I* > 2σ(*I*)	intensity decay: 1%

### Refinement

**Table:**

Refinement on *F*^2^	Primary atom site location: structure-invariant direct methods
Least-squares matrix: full	Secondary atom site location: difference Fourier map
*R*[*F*^2^ > 2σ(*F*^2^)] = 0.060	Hydrogen site location: inferred from neighbouring sites
*wR*(*F*^2^) = 0.187	H-atom parameters constrained
*S* = 1.00	*w* = 1/[σ^2^(*F*_o_^2^) + (0.1*P*)^2^ + 0.090*P*] where *P* = (*F*_o_^2^ + 2*F*_c_^2^)/3
4436 reflections	(Δ/σ)_max_ < 0.001
307 parameters	Δρ_max_ = 0.58 e Å^−^^3^
8 restraints	Δρ_min_ = −0.24 e Å^−^^3^

### Special details

**Table:**

Geometry. All e.s.d.'s (except the e.s.d. in the dihedral angle between two l.s. planes) are estimated using the full covariance matrix. The cell e.s.d.'s are taken into account individually in the estimation of e.s.d.'s in distances, angles and torsion angles; correlations between e.s.d.'s in cell parameters are only used when they are defined by crystal symmetry. An approximate (isotropic) treatment of cell e.s.d.'s is used for estimating e.s.d.'s involving l.s. planes.
Refinement. Refinement of *F*^2^ against ALL reflections. The weighted *R*-factor *wR* and goodness of fit *S* are based on *F*^2^, conventional *R*-factors *R* are based on *F*, with *F* set to zero for negative *F*^2^. The threshold expression of *F*^2^ > σ(*F*^2^) is used only for calculating *R*-factors(gt) *etc*. and is not relevant to the choice of reflections for refinement. *R*-factors based on *F*^2^ are statistically about twice as large as those based on *F*, and *R*- factors based on ALL data will be even larger.

### Fractional atomic coordinates and isotropic or equivalent isotropic displacement parameters (Å^2^)

**Table:**

	*x*	*y*	*z*	*U*_iso_*/*U*_eq_	
N1	0.7132 (2)	0.55000 (19)	0.14805 (11)	0.0548 (6)	
O1	0.56644 (19)	0.84300 (16)	−0.00661 (11)	0.0693 (6)	
F1	1.1646 (2)	0.3281 (2)	0.36341 (10)	0.1089 (8)	
C1	0.8741 (3)	0.5005 (3)	0.31912 (15)	0.0699 (8)	
H1A	0.8292	0.5566	0.3382	0.084*	
N2	0.7122 (2)	0.71486 (19)	0.04413 (12)	0.0616 (6)	
O2	1.0363 (2)	1.4223 (2)	0.07819 (13)	0.0929 (7)	
F2	0.3037 (2)	0.1832 (2)	0.22049 (13)	0.1311 (10)	
C2	0.9889 (3)	0.4553 (3)	0.35609 (16)	0.0807 (10)	
H2A	1.0218	0.4806	0.3995	0.097*	
C3	1.0521 (3)	0.3731 (3)	0.32737 (17)	0.0744 (9)	
C4	1.0074 (3)	0.3339 (3)	0.26406 (16)	0.0733 (9)	
H4A	1.0528	0.2776	0.2455	0.088*	
C5	0.8921 (3)	0.3803 (3)	0.22795 (15)	0.0696 (8)	
H5A	0.8596	0.3541	0.1847	0.084*	
C6	0.8251 (3)	0.4642 (2)	0.25486 (14)	0.0574 (7)	
C7	0.5877 (3)	0.4256 (3)	0.21433 (14)	0.0620 (8)	
C8	0.5638 (3)	0.3419 (3)	0.16599 (16)	0.0692 (8)	
H8A	0.6143	0.3401	0.1318	0.083*	
C9	0.4676 (3)	0.2613 (3)	0.16697 (19)	0.0814 (10)	
H9A	0.4505	0.2074	0.1332	0.098*	
C10	0.3985 (3)	0.2628 (4)	0.2187 (2)	0.0887 (12)	
C11	0.4166 (4)	0.3418 (4)	0.2671 (2)	0.1001 (13)	
H11A	0.3666	0.3410	0.3015	0.120*	
C12	0.5114 (3)	0.4248 (4)	0.26493 (17)	0.0819 (10)	
H12A	0.5239	0.4805	0.2978	0.098*	
C13	0.6959 (3)	0.5127 (3)	0.21574 (13)	0.0617 (8)	
H13A	0.6720	0.5784	0.2404	0.074*	
C14	0.5897 (3)	0.5936 (2)	0.10899 (14)	0.0616 (8)	
H14A	0.5601	0.6579	0.1321	0.074*	
H14B	0.5214	0.5363	0.1053	0.074*	
C15	0.6098 (3)	0.6283 (2)	0.03945 (15)	0.0648 (8)	
H15A	0.6360	0.5635	0.0156	0.078*	
H15B	0.5271	0.6570	0.0142	0.078*	
C16	0.8365 (3)	0.6765 (3)	0.08475 (15)	0.0651 (8)	
H16A	0.9008	0.7372	0.0898	0.078*	
H16B	0.8721	0.6147	0.0620	0.078*	
C17	0.8146 (3)	0.6384 (2)	0.15362 (14)	0.0623 (8)	
H17A	0.8975	0.6101	0.1790	0.075*	
H17B	0.7869	0.7019	0.1779	0.075*	
C18	0.6794 (3)	0.8217 (2)	0.02320 (13)	0.0547 (7)	
C19	0.7819 (3)	0.9100 (2)	0.03716 (14)	0.0587 (7)	
H19A	0.8700	0.8902	0.0524	0.070*	
C20	0.7490 (3)	1.0179 (2)	0.02810 (13)	0.0584 (7)	
H20A	0.6601	1.0318	0.0109	0.070*	
C21	0.8321 (3)	1.1167 (2)	0.04123 (14)	0.0568 (7)	
C22	0.7743 (3)	1.2220 (2)	0.02963 (16)	0.0726 (9)	
H22A	0.6839	1.2261	0.0124	0.087*	
C23	0.8435 (4)	1.3196 (3)	0.04231 (18)	0.0823 (9)	
H23A	0.8003	1.3884	0.0344	0.099*	
C24	0.9746 (4)	1.3163 (3)	0.06624 (15)	0.0720 (8)	
C25	1.0407 (3)	1.2158 (3)	0.07832 (16)	0.0734 (8)	
H25A	1.1316	1.2141	0.0944	0.088*	
C26	0.9671 (3)	1.1144 (3)	0.06569 (15)	0.0724 (9)	
H26A	1.0102	1.0457	0.0740	0.087*	
C27	1.1649 (3)	1.4225 (3)	0.11139 (18)	0.0893 (10)	
H27A	1.2226	1.3913	0.0827	0.107*	
H27B	1.1727	1.3767	0.1517	0.107*	
C28	1.2053 (4)	1.5424 (3)	0.1301 (2)	0.1015 (12)	
H28A	1.2952	1.5435	0.1540	0.152*	
H28B	1.1475	1.5729	0.1584	0.152*	
H28C	1.1990	1.5869	0.0899	0.152*	

### Atomic displacement parameters (Å^2^)

**Table:**

	*U*^11^	*U*^22^	*U*^33^	*U*^12^	*U*^13^	*U*^23^
N1	0.0533 (13)	0.0566 (14)	0.0538 (13)	0.0010 (11)	0.0074 (10)	0.0031 (11)
O1	0.0608 (12)	0.0615 (13)	0.0772 (14)	0.0057 (10)	−0.0114 (10)	0.0069 (10)
F1	0.0855 (14)	0.150 (2)	0.0849 (14)	0.0211 (14)	−0.0016 (11)	0.0355 (14)
C1	0.081 (2)	0.075 (2)	0.0536 (18)	−0.0019 (18)	0.0120 (16)	−0.0040 (16)
N2	0.0577 (14)	0.0526 (14)	0.0705 (16)	0.0017 (12)	0.0001 (12)	0.0116 (12)
O2	0.0900 (16)	0.0816 (13)	0.1009 (18)	−0.0015 (12)	−0.0001 (14)	0.0015 (14)
F2	0.0902 (15)	0.152 (2)	0.145 (2)	−0.0403 (16)	0.0027 (14)	0.0606 (17)
C2	0.084 (2)	0.106 (3)	0.0476 (18)	−0.004 (2)	0.0005 (17)	0.0058 (18)
C3	0.062 (2)	0.097 (3)	0.064 (2)	0.0043 (19)	0.0075 (17)	0.0287 (19)
C4	0.073 (2)	0.086 (2)	0.063 (2)	0.0126 (18)	0.0179 (17)	0.0065 (18)
C5	0.071 (2)	0.085 (2)	0.0518 (17)	0.0085 (18)	0.0091 (15)	−0.0014 (16)
C6	0.0575 (17)	0.0670 (19)	0.0490 (16)	−0.0017 (15)	0.0127 (13)	0.0056 (14)
C7	0.0571 (17)	0.076 (2)	0.0537 (17)	0.0067 (16)	0.0131 (14)	0.0116 (16)
C8	0.068 (2)	0.070 (2)	0.071 (2)	0.0031 (17)	0.0151 (16)	0.0110 (17)
C9	0.074 (2)	0.072 (2)	0.091 (3)	−0.0020 (19)	−0.006 (2)	0.0147 (19)
C10	0.062 (2)	0.109 (3)	0.092 (3)	−0.013 (2)	0.004 (2)	0.039 (2)
C11	0.064 (2)	0.156 (4)	0.084 (3)	−0.005 (3)	0.022 (2)	0.037 (3)
C12	0.069 (2)	0.115 (3)	0.063 (2)	0.003 (2)	0.0155 (17)	0.0078 (19)
C13	0.0646 (18)	0.0692 (19)	0.0530 (17)	0.0027 (16)	0.0144 (14)	−0.0034 (15)
C14	0.0557 (17)	0.0574 (18)	0.070 (2)	0.0026 (14)	0.0063 (14)	0.0019 (15)
C15	0.0612 (18)	0.0597 (18)	0.0684 (19)	−0.0020 (15)	−0.0025 (15)	0.0067 (15)
C16	0.0544 (17)	0.0619 (18)	0.077 (2)	0.0050 (15)	0.0059 (15)	0.0160 (16)
C17	0.0557 (17)	0.0654 (19)	0.0620 (18)	0.0022 (15)	−0.0007 (14)	0.0033 (15)
C18	0.0622 (17)	0.0511 (16)	0.0503 (16)	0.0084 (14)	0.0086 (13)	0.0030 (13)
C19	0.0533 (16)	0.0616 (19)	0.0610 (18)	0.0023 (15)	0.0090 (14)	0.0068 (14)
C20	0.0637 (18)	0.0586 (19)	0.0500 (16)	0.0062 (15)	0.0022 (13)	0.0006 (13)
C21	0.0626 (18)	0.0588 (18)	0.0487 (16)	0.0012 (15)	0.0089 (13)	−0.0020 (13)
C22	0.082 (2)	0.058 (2)	0.073 (2)	−0.0019 (18)	0.0001 (17)	−0.0073 (16)
C23	0.0947 (18)	0.061 (2)	0.085 (2)	0.0049 (15)	−0.0021 (19)	−0.0068 (17)
C24	0.0920 (18)	0.0696 (16)	0.0567 (18)	−0.0033 (14)	0.0191 (16)	−0.0031 (15)
C25	0.0616 (17)	0.0872 (16)	0.072 (2)	−0.0051 (15)	0.0132 (16)	−0.0012 (17)
C26	0.073 (2)	0.071 (2)	0.073 (2)	0.0035 (18)	0.0149 (17)	−0.0009 (17)
C27	0.077 (2)	0.111 (2)	0.082 (2)	−0.013 (2)	0.020 (2)	−0.004 (2)
C28	0.085 (2)	0.103 (2)	0.112 (3)	−0.016 (2)	0.007 (2)	−0.015 (2)

### Geometric parameters (Å, °)

**Table:**

N1—C14	1.462 (3)	C13—H13A	0.9800
N1—C17	1.468 (3)	C14—C15	1.509 (4)
N1—C13	1.474 (3)	C14—H14A	0.9700
O1—C18	1.232 (3)	C14—H14B	0.9700
F1—C3	1.359 (3)	C15—H15A	0.9700
C1—C6	1.374 (4)	C15—H15B	0.9700
C1—C2	1.385 (4)	C16—C17	1.513 (4)
C1—H1A	0.9300	C16—H16A	0.9700
N2—C18	1.362 (3)	C16—H16B	0.9700
N2—C16	1.460 (3)	C17—H17A	0.9700
N2—C15	1.460 (3)	C17—H17B	0.9700
O2—C27	1.368 (4)	C18—C19	1.477 (4)
O2—C24	1.412 (4)	C19—C20	1.332 (4)
F2—C10	1.361 (4)	C19—H19A	0.9300
C2—C3	1.356 (5)	C20—C21	1.447 (4)
C2—H2A	0.9300	C20—H20A	0.9300
C3—C4	1.360 (4)	C21—C26	1.383 (4)
C4—C5	1.387 (4)	C21—C22	1.388 (4)
C4—H4A	0.9300	C22—C23	1.361 (4)
C5—C6	1.375 (4)	C22—H22A	0.9300
C5—H5A	0.9300	C23—C24	1.344 (5)
C6—C13	1.528 (4)	C23—H23A	0.9300
C7—C8	1.384 (4)	C24—C25	1.374 (4)
C7—C12	1.388 (4)	C25—C26	1.421 (4)
C7—C13	1.513 (4)	C25—H25A	0.9300
C8—C9	1.376 (4)	C26—H26A	0.9300
C8—H8A	0.9300	C27—C28	1.515 (5)
C9—C10	1.357 (5)	C27—H27A	0.9700
C9—H9A	0.9300	C27—H27B	0.9700
C10—C11	1.344 (6)	C28—H28A	0.9600
C11—C12	1.389 (5)	C28—H28B	0.9600
C11—H11A	0.9300	C28—H28C	0.9600
C12—H12A	0.9300		
			
C14—N1—C17	108.6 (2)	N2—C15—H15A	109.6
C14—N1—C13	111.9 (2)	C14—C15—H15A	109.6
C17—N1—C13	110.0 (2)	N2—C15—H15B	109.6
C6—C1—C2	121.4 (3)	C14—C15—H15B	109.6
C6—C1—H1A	119.3	H15A—C15—H15B	108.1
C2—C1—H1A	119.3	N2—C16—C17	110.7 (2)
C18—N2—C16	127.5 (2)	N2—C16—H16A	109.5
C18—N2—C15	120.2 (2)	C17—C16—H16A	109.5
C16—N2—C15	111.1 (2)	N2—C16—H16B	109.5
C27—O2—C24	116.6 (3)	C17—C16—H16B	109.5
C3—C2—C1	118.2 (3)	H16A—C16—H16B	108.1
C3—C2—H2A	120.9	N1—C17—C16	111.1 (2)
C1—C2—H2A	120.9	N1—C17—H17A	109.4
C2—C3—F1	118.6 (3)	C16—C17—H17A	109.4
C2—C3—C4	122.7 (3)	N1—C17—H17B	109.4
F1—C3—C4	118.8 (3)	C16—C17—H17B	109.4
C3—C4—C5	118.2 (3)	H17A—C17—H17B	108.0
C3—C4—H4A	120.9	O1—C18—N2	120.2 (3)
C5—C4—H4A	120.9	O1—C18—C19	121.4 (2)
C6—C5—C4	121.2 (3)	N2—C18—C19	118.5 (2)
C6—C5—H5A	119.4	C20—C19—C18	120.4 (3)
C4—C5—H5A	119.4	C20—C19—H19A	119.8
C1—C6—C5	118.3 (3)	C18—C19—H19A	119.8
C1—C6—C13	120.5 (3)	C19—C20—C21	129.2 (3)
C5—C6—C13	121.1 (3)	C19—C20—H20A	115.4
C8—C7—C12	117.3 (3)	C21—C20—H20A	115.4
C8—C7—C13	123.0 (3)	C26—C21—C22	116.6 (3)
C12—C7—C13	119.6 (3)	C26—C21—C20	124.5 (3)
C9—C8—C7	122.0 (3)	C22—C21—C20	118.9 (3)
C9—C8—H8A	119.0	C23—C22—C21	123.1 (3)
C7—C8—H8A	119.0	C23—C22—H22A	118.4
C10—C9—C8	118.1 (4)	C21—C22—H22A	118.4
C10—C9—H9A	120.9	C24—C23—C22	119.7 (3)
C8—C9—H9A	120.9	C24—C23—H23A	120.1
C11—C10—C9	122.8 (4)	C22—C23—H23A	120.1
C11—C10—F2	118.4 (4)	C23—C24—C25	121.2 (3)
C9—C10—F2	118.7 (4)	C23—C24—O2	115.0 (3)
C10—C11—C12	118.8 (4)	C25—C24—O2	123.8 (3)
C10—C11—H11A	120.6	C24—C25—C26	118.6 (3)
C12—C11—H11A	120.6	C24—C25—H25A	120.7
C7—C12—C11	120.9 (4)	C26—C25—H25A	120.7
C7—C12—H12A	119.6	C21—C26—C25	120.8 (3)
C11—C12—H12A	119.6	C21—C26—H26A	119.6
N1—C13—C7	113.2 (2)	C25—C26—H26A	119.6
N1—C13—C6	111.0 (2)	O2—C27—C28	108.7 (3)
C7—C13—C6	108.4 (2)	O2—C27—H27A	109.9
N1—C13—H13A	108.0	C28—C27—H27A	109.9
C7—C13—H13A	108.0	O2—C27—H27B	109.9
C6—C13—H13A	108.0	C28—C27—H27B	109.9
N1—C14—C15	110.6 (2)	H27A—C27—H27B	108.3
N1—C14—H14A	109.5	C27—C28—H28A	109.5
C15—C14—H14A	109.5	C27—C28—H28B	109.5
N1—C14—H14B	109.5	H28A—C28—H28B	109.5
C15—C14—H14B	109.5	C27—C28—H28C	109.5
H14A—C14—H14B	108.1	H28A—C28—H28C	109.5
N2—C15—C14	110.2 (2)	H28B—C28—H28C	109.5
			
C6—C1—C2—C3	−0.4 (5)	C17—N1—C14—C15	59.8 (3)
C1—C2—C3—F1	−179.8 (3)	C13—N1—C14—C15	−178.5 (2)
C1—C2—C3—C4	0.1 (5)	C18—N2—C15—C14	−111.9 (3)
C2—C3—C4—C5	−0.2 (5)	C16—N2—C15—C14	56.4 (3)
F1—C3—C4—C5	179.7 (3)	N1—C14—C15—N2	−59.3 (3)
C3—C4—C5—C6	0.6 (5)	C18—N2—C16—C17	112.2 (3)
C2—C1—C6—C5	0.8 (5)	C15—N2—C16—C17	−55.1 (3)
C2—C1—C6—C13	178.1 (3)	C14—N1—C17—C16	−58.4 (3)
C4—C5—C6—C1	−0.8 (5)	C13—N1—C17—C16	178.8 (2)
C4—C5—C6—C13	−178.2 (3)	N2—C16—C17—N1	56.5 (3)
C12—C7—C8—C9	0.7 (4)	C16—N2—C18—O1	−174.4 (3)
C13—C7—C8—C9	177.8 (3)	C15—N2—C18—O1	−8.1 (4)
C7—C8—C9—C10	−2.5 (5)	C16—N2—C18—C19	5.6 (4)
C8—C9—C10—C11	2.6 (5)	C15—N2—C18—C19	171.8 (2)
C8—C9—C10—F2	−179.6 (3)	O1—C18—C19—C20	11.4 (4)
C9—C10—C11—C12	−1.0 (6)	N2—C18—C19—C20	−168.6 (3)
F2—C10—C11—C12	−178.8 (3)	C18—C19—C20—C21	177.2 (3)
C8—C7—C12—C11	1.0 (5)	C19—C20—C21—C26	1.1 (5)
C13—C7—C12—C11	−176.2 (3)	C19—C20—C21—C22	−178.0 (3)
C10—C11—C12—C7	−0.9 (5)	C26—C21—C22—C23	−1.2 (5)
C14—N1—C13—C7	56.2 (3)	C20—C21—C22—C23	178.1 (3)
C17—N1—C13—C7	177.0 (2)	C21—C22—C23—C24	1.1 (5)
C14—N1—C13—C6	178.3 (2)	C22—C23—C24—C25	−0.1 (5)
C17—N1—C13—C6	−60.8 (3)	C22—C23—C24—O2	−179.4 (3)
C8—C7—C13—N1	37.2 (4)	C27—O2—C24—C23	171.0 (3)
C12—C7—C13—N1	−145.8 (3)	C27—O2—C24—C25	−8.4 (4)
C8—C7—C13—C6	−86.5 (3)	C23—C24—C25—C26	−0.7 (5)
C12—C7—C13—C6	90.6 (3)	O2—C24—C25—C26	178.6 (3)
C1—C6—C13—N1	129.6 (3)	C22—C21—C26—C25	0.4 (4)
C5—C6—C13—N1	−53.1 (4)	C20—C21—C26—C25	−178.8 (3)
C1—C6—C13—C7	−105.5 (3)	C24—C25—C26—C21	0.5 (4)
C5—C6—C13—C7	71.8 (3)	C24—O2—C27—C28	−170.2 (3)

### Hydrogen-bond geometry (Å, °)

**Table:**

*D*—H···*A*	*D*—H	H···*A*	*D*···*A*	*D*—H···*A*
C9—H9A···O1^i^	0.93	2.60	3.422 (4)	148

Symmetry codes: (i) −*x*+1, −*y*+1, −*z*.
